# Time trends in demographic characteristics of participants and outcome measures in Parkinson’s disease research: A 19-year single-center experience

**DOI:** 10.1016/j.prdoa.2023.100185

**Published:** 2023-01-27

**Authors:** Bart R. Maas, Bastiaan R. Bloem, Yoav Ben-Shlomo, Luc J.W. Evers, Rick C. Helmich, Johanna G. Kalf, Marjolein A. van der Marck, Marjan J. Meinders, Alice Nieuwboer, Maarten J. Nijkrake, Jorik Nonnekes, Bart Post, Ingrid H.W.M. Sturkenboom, Marcel M. Verbeek, Nienke M. de Vries, Bart van de Warrenburg, Tessa van de Zande, Marten Munneke, Sirwan K.L. Darweesh

**Affiliations:** a Department of Neurology, Radboud University Medical Centre, Donders Institute for Brain, Cognition and Behavior, Nijmegen, The Netherlands; b Department of Population Health Sciences, Bristol Medical School, University of Bristol, Bristol, UK; c Department of Rehabilitation, Radboud University Medical Centre, Donders Institute for Brain, Cognition and Behavior, Nijmegen, The Netherlands; d Department of Geriatrics, Radboud Institute for Health Sciences, Radboud University Medical Center, Nijmegen, The Netherlands; e Scientific Center for Quality of Healthcare, Radboud Institute for Health Sciences, Radboud University Medical Center, Nijmegen, The Netherlands; f Department of Rehabilitation Sciences, Neurorehabilitation Research Group, KU Leuven, Leuven, Belgium; g Department of Rehabilitation, Sint Maartenskliniek, Nijmegen, The Netherlands; h Department of Laboratory Medicine, Radboud University Medical Center, Nijmegen, The Netherlands

**Keywords:** Parkinson's disease, Parkinsonism, Time trends, Participant characteristics, Demographics, Outcome measure, Diversity, Representative, Underrepresentation, Gender, Sex, Females, Age, Elderly, Young-onset, Ehtnicity, Non-motor symptoms

## Abstract

**Background:**

Females, people with young-onset PD and older individuals, and non-white populations are historically underrepresented in clinical Parkinson’s disease (PD) research. Furthermore, research traditionally focused predominantly on motor symptoms of PD. Including a representative and diverse group of people with PD and also studying non-motor symptoms is warranted to better understand heterogeneity in PD and to generalize research findings.

**Objective:**

This project aimed to determine whether, within a consecutive series of PD studies performed within a single center in the Netherlands: (1) the proportion of included females, mean age and proportion of native Dutch people changed over time; and 2) reports of the ethnicity of participants and the proportion of studies with non-motor outcomes changed over time.

**Methods:**

Characteristics of participants and non-motor outcomes were analyzed using a unique dataset of summary statistics of studies with a large number of participants conducted at a single center during a 19-year period (2003–2021).

**Results:**

Results indicate no relationship between calendar time and proportion of females (mean 39 %), mean age (66 years), proportion of studies that reported ethnicity, and proportion of native Dutch people in studies (range 97–100 %). The proportion of participants in whom non-motor symptoms were assessed increased, but this difference was consistent with chance.

**Conclusion:**

Study participants in this center reflect the PD population in the Netherlands in terms of sex, but older individuals and non-native Dutch individuals are under-represented. We have still a lot to do in ensuring adequate representation and diversity in PD patients within our research.

## Highlights

• Parkinson’s disease studies historically focus on selective subgroups and motor symptoms.
• We examined 33 Parkinson’s disease studies at a single center between 2003 and 2021.
• We observed no temporal changes in adequate representation by sex, age or ethnicity.
• It was equivocal whether assessment of non-motor symptoms increased over time.
• Improved representation and non-motor assessments are warranted in future studies.

## 1 Introduction

Parkinson’s disease (PD) is a common neurodegenerative disease, affecting both males and females across a range of ages, socioeconomic statuses and racial and ethnic backgrounds. Despite this heterogeneity, certain subgroups of people are historically underrepresented in clinical PD research, in particular females, both people with young-onset PD and older individuals, individuals with atypical parkinsonism, people with a lower socioeconomic status (SES), individuals with cognitive dysfunctions or interfering comorbidity, people with advanced PD and non-white populations [1]. This selective inclusion is disconcerting, because outcomes may well be different among people with PD (PwP) in various underrepresented groups [1].

Over the last decade there has been an increased attention to non-motor symptoms (NMS) including sleep disturbances, gastrointestinal dysfunction, bladder dysfunction, and fatigue [2]. These NMS have a larger impact on the health-related quality of life of PwP than motor symptoms [3]. Yet, observational research has still mainly focused on the motor symptoms of PD [4]. Inclusion of a diverse group of PwP and also studying NMS is warranted to better understand the heterogeneity in PD and to generalize research findings.

Although awareness about including a more diverse group of PwP and focus on non-motor outcomes is growing in PD research [5], there are no studies available that investigated whether this has led to more diversity in study participants and more assessment of non-motor outcomes in PD studies over time. Therefore, we analyzed summary statistics of studies with large numbers of participants conducted at a single center during a 19-year period (2003–2021). We hypothesized increasing proportions of historically underrepresented groups and an increase of assessment of non-motor outcome measures over time.

## 2 Methods

### 2.1 Study design

The Radboudumc Center of Expertise for Parkinson & Movement Disorders based in Nijmegen, Netherlands, has a long-standing history of conducting PD research. In the current paper, participant characteristics and non-motor outcomes of all studies were investigated, conducted at this single center over a period of 19 years (2003–2021).

### 2.2 Outcome measures

Outcome variables in the current paper are the proportion of females, mean age, proportion of native Dutch people, number of studies that reported participants’ ethnicity, and number of studies with NMS as outcome measure. We allowed studies to use different ways to measure ethnicity of their participants, e.g. country of birth or self-reported ethnic group. Although these are not the same, for our analysis we assume they both capture ethnicity. The year in which studies started recruiting their participants, rather than publication, was defined as calendar time.

### 2.3 Statistical analysis

We investigated associations between calendar time and both proportion of females and mean age using random effects inverse-variance meta-analysis. This was done both in an univariable and multivariable meta-regression approach, correcting for age and sex, unless it was the outcome variable. We also investigated whether outcome measures differed between three time periods (2003–2009, 2010–2015 and 2016–2021), using a Wilcoxon rank sum test. This was done both weighted and unweighted for sample size of the study. Furthermore, we performed a sensitivity analysis to investigate whether the number of studies with a high proportion of females (>50 %) and studies with a high (>70 years) or low (<60 years) mean age differed between the time periods (2003–2009, 2010–2015 and 2016–2021) using a Wilcoxon rank sum test. Statistical analyses were performed using R software.

## 3 Results

### 3.1 Descriptive analysis

We identified 33 eligible studies for our analysis, of which 12 studies were both completed and published. Ten studies were not yet completed but had published their protocol (see Supplementary material *A*).

### 3.2 Sex

**Fig. 1:**
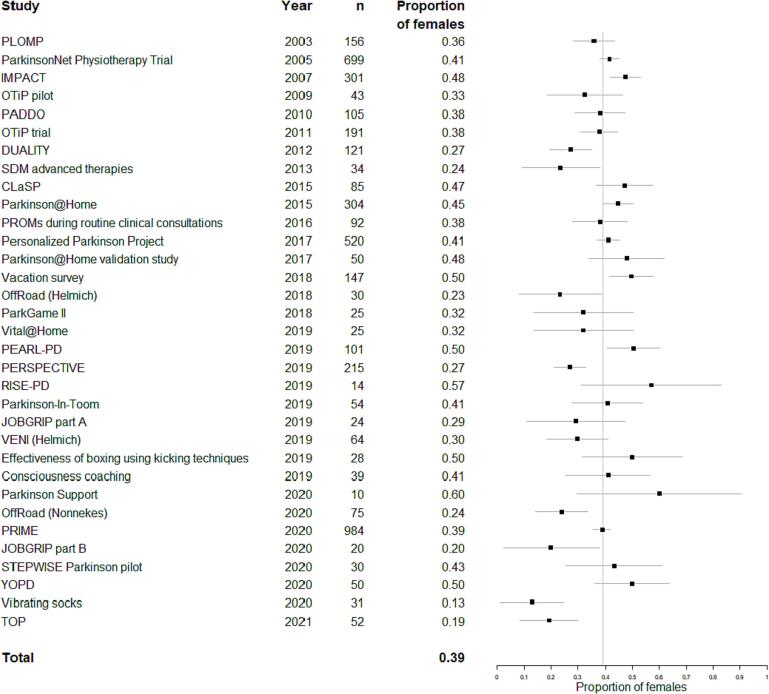
Proportion of females. *Forest plot showing proportion of females and 95% confidence intervals for each study performed at our center. The vertical grey line indicates the mean proportion of females (weighted for study size) in studies in our center over the entire study period. The number of participants in each study is shown as well.*

Fig. 1 shows that the proportion of females in PD studies (39 %) did not change over time (β = −0.004, p = 0.27), even after correction for age (β = −0.004, p = 0.31). Of the 22 published studies or protocols, three studies used the term sex and twelve used the term gender, but none of them investigated the gender identity of their participants.

### 3.3 Age

**Fig. 2:**
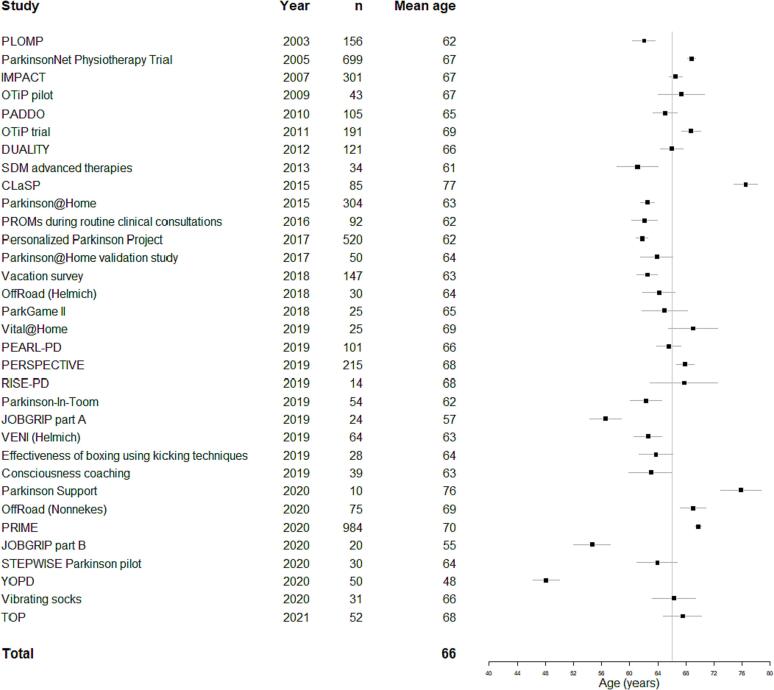
Age over time. *Forest plot showing the mean age and 95% confidence intervals of participants per study performed at our center. The vertical grey line indicates the overall mean age of participants (weighted for study size) in all studies in our center. The number of participants in each study is shown as well.*

Fig. 2 shows that the mean age of study participants (66 years) did not change over time (β = −0.159, p = 0.61), even after correction for proportion of females (β = −0.156, p = 0.62). Mean age of PD participants also had no relation with mean age at death in the Netherlands (β = −1.333, p = 0.43) which was surprising given the increasing number of older PwP.

### 3.4 Ethnicity

Only eight out of 33 studies reported the ethnicity of study participants (Supplementary material *A*). The number of studies reporting the ethnicity of their participants did not change over time (Supplementary material *B*). The proportion of native Dutch people in studies in these eight studies did also not change over time (between 97 and 100 %).

### 3.5 Non-motor symptom measures over time

There were 11 out of 33 studies that assessed NMS as an outcome measure (Supplementary material *A*). We observed an increasing trend over time in the proportion of participants of whom NMS were assessed, from 25 % in 2003–2009 to 64 % in 2016–2021, although this difference was consistent with chance (Supplementary material *B*).

### 3.6 Sensitivity analyses

Sensitivity analyses showed that studies with a proportion of females similar or >50 %, a mean age below 60 and a mean age above 70 did not statistically differ between timeframes (2003–2009, 2010–2015 and 2016–2021).

## 4 Discussion

The main finding of this retrospective observational study is that we did not observe a relationship between calendar time with research participant characteristics and outcome measures in studies on PD conducted at a single tertiary center in the Netherlands, which is somewhat surprising and requires further attention.

Before we interpret our results, we must consider the external validity of our sample, as an indicator of the generalizability. A single-center analysis has several advantages. First, participants are recruited from one country and within a single healthcare system, which makes the spatial parameter small and allows us to better determine the influence of the temporal parameter in specified outcomes. This is, however, also a limitation, because the results may not be generalizable to other counties, in particular the temporal data on participant characteristics. Second, we were able to use very recent data of unfinished and unpublished studies, which is not possible in a meta-analysis of published data only. Third, the lead in our study center did not change over the past 19 years, which may have reduced the change over time in person-specific interests of the research group, although the interests of our center’s lead may in itself have evolved over time. Aside from the external validity of our sample, we recognize that a limitation of the current study is that only summary statistic data were available and we are therefore not able to analyze individual participant strata of for example early vs late onset PD or males vs females. Furthermore, we were unable to analyze underrepresented groups in PD research such as those with cognitive dysfunctions, comorbidities and people with advanced PD or to analyze severity of motor symptoms, because the these clinical characteristics were not available for us. We also acknowledge that participant characteristics and outcome measures depend on the research questions, which are rather center specific. For example, research on early onset PD includes only younger individuals and therefore we performed several sensitivity analyses.

To assess whether our study population is a good reflection of the total PD population in the Netherlands, we compared the proportion of females, the mean age and proportion of native Dutch people in our sample with external estimates. A systematic review and meta-analysis reported that 45 % of the PwP is female in Europe/North America/Australia [6]. Another paper found that females are underrepresented in PD clinical trials, namely 40 % of participants is female in PD randomized controlled trials [7]. Two large studies with data of more than 4,000 [8] and 51,000 [9] PwP in the Netherlands using medical claims data, both reported the proportion of females as 42 %. The proportion of females in our analysis (39 %) is close to these external estimates, suggesting that the proportion of females appears to have been roughly representative of the spectrum of PwP. We also critically appraised which terminology was used to report the proportion of females in our studies. “Gender” differences are rooted in different expressions of identity, adherence to norms, and socially defined behaviors [10]. “Sex” differences are based on biological variations due to differences in genetics, hormones, and physiology. Although twelve studies within our center used the term gender, none of them assessed gender.

We observed that the mean age within most PD studies is 66 years, which is 6 years lower than found in a medical claims data study (n > 51,000) which reported an average age of 72 years [9]. Under-representation of older patients is further supported by the observation that the mean age of death of people in the Netherlands increased by 3.3 years (75.6 years in 2003 to 78.9 years in 2020 [11] but this was not reflected in an increase in the mean age of participants in PD studies. Possible reasons for this under-representation include (i) some studies specifically recruited people with young-onset PD, or individuals with a recent diagnosis (≤3 years), though the mean age was still too low even if we excluded young onset studies. (ii) A more likely reason is selection bias as much of our research were intervention studies and these may either be more attractive for fitter and younger individuals or actually exclude frailer or cognitively impaired individuals who will usually be older.

The distribution of ethnicity in the Dutch PD population is hard to estimate, because this characteristic of participants is often not reported in medical research. We also observed this in our analysis. Only eight out of 33 studies reported the ethnicity of their study participants. In all eight studies, the proportion of native Dutch people was above 97 %. In 2003, 19 % of the general Dutch population had a migration background and in 2021 this was 25 %, meaning that they or one or both of their parents were not born in the Netherlands [12]. A non-Dutch ethnicity is different from a migration background, because ethnicity includes not only the place of birth, but also cultural background. We assume that the age-adjusted prevalence of PD is similar across people with different backgrounds, however due to the younger age distribution of migrant populations it is likely that there will be fewer patients with PD from a minority ethnic group. However, the very high figure of 97 % plus for native Dutch participants is highly suggestive that our research population under-represents non-native Dutch PD populations. This subject has recently received more attention in the Netherlands, among other things due to demands from grant agencies, but in actual research practice, it still appears to be lagging behind. This may be caused by differences in language, culture and how people look to the disease or feel about participating in research. Reaching out to these underserved populations can be challenging but future studies must pay more attention to their methods of participant recruitment and tailor this to different ethnic groups if we are to do better. Simply translating recruitment materials into different languages, whilst helpful, is likely to be inadequate and a multi-pronged approach using clinical staff, relevant community leaders and influencers as well as appropriate social media will be necessary.

Given the clinical heterogeneity of PD, it is important that studies not only investigate motor symptoms but also NMS. We did observe an increase in studies that included NMS as an outcome measure in our center. This modest growth is explained in part by a greater awareness of NMS, but also by the retrospective character of the present study: some NMS tools did not exist in 2003. However, even in the most recent time period around a third of studies did not focus on NMS even though they are a key determinant of quality of life in PwP. Furthermore, facilitating clinical research closer to real life of PwP by for example continuous monitoring using wearable sensors may have added value.

In conclusion, the PD research community still has a lot to do in ensuring adequate representation and diversity in PD patients recruited into clinical research, assuming our results are generalizable of research conducted in other high-income countries. It would be helpful to see if other centers or countries find similar results or do a better job in ensuring good representation. Further research should explore and evaluate how we can remove the barriers and implement facilitators of equitable access to the research experience for all patients with PD who wish to help the research endeavor.

## 5 Ethical compliance statement

This study was performed in accordance with The Code of Ethics of the World Medical Association (Declaration of Helsinki). No ethical institutional approval was required since this study only entailed analyses of summary statistics, not of individual participant data.

## Author contributions

All authors played a substantial role in interpretation of data and reviewing manuscript. BRM and SKLD contributed to the design, conceptualization of the study, were responsible for data acquisition and were responsible for statistical analysis and validation. BRM and SKLD drafted the manuscript and all other authors were responsible for reviewing manuscript and providing critical intellectual input.

## Declaration of Competing Interest

The authors declare that they have no known competing financial interests or personal relationships that could have appeared to influence the work reported in this paper.
