# Supplementary material for: Time trends in demographic characteristics of participants and outcome measures in Parkinson’s disease research: A 19-year single-center experience
Source: Clin Park Relat Disord. 2023 Jan 27;8:100185. doi: 10.1016/j.prdoa.2023.100185 (PMC9923175; doi:10.1016/j.prdoa.2023.100185)
Supplement: Supplementary Data 1 [file mmc1.docx]

**Supplementary material**

Supplementary material A. Overview of studies

| Study | Reference | Year start inclusion | N | Diagnosis | Age | Proportion females | Ethnicity reported | Proportion of native Dutch people | NMS as outcome |
| --- | --- | --- | --- | --- | --- | --- | --- | --- | --- |
|  |  |  |  |  | Mean | % | Yes/no | % | Yes/no |
| **Mean for all studies^a^** |  |  |  |  | **66.2** | **39.4** |  |  |  |
| PLOMP | A | 2003 | 156 | PD + AP | 62 | 35.9 | Yes | 99 | No |
| ParkinsonNet Physiotherapy Trial | B | 2005 | 699 | PD | 68.8 | 41.5 | No |  | No |
| IMPACT | C | 2007 | 301 | PD | 66.5 | 47.5 | No |  | Yes |
| OTiP pilot | D | 2009 | 43 | PD | 67.3 | 32.6 | No |  | No |
| PADDO | E | 2010 | 105 | PD + AP | 65 | 38.1 | Yes | 100 | No |
| OTiP trial | F^b^ | 2011 | 191 | PD | 68.7 | 37.7 | No |  | Yes |
| DUALITY | G | 2012 | 121 | PD | 65.9 | 27.3 | No |  | No |
| SDM advanced therapies | H^b^ | 2013 | 34 | PD | 61.1 | 23.5 | No |  | No |
| CLaSP | I | 2015 | 85 | PD | 76.5 | 47.1 | No |  | Yes |
| Parkinson@Home | J | 2015 | 304 | PD | 62.5 | 44.7 | No |  | No |
| PROMs during routine clinical consultations | K | 2016 | 92 | PD | 62.1 | 38.0 | Yes | 97 | No |
| Personalized Parkinson Project | L^b^ | 2017 | 520 | PD | 61.7 | 41.2 | Yes |  | Yes |
| Parkinson@Home validation study | M | 2017 | 25 | PD | 63.8 | 48 | No |  | No |
| Vacation survey | Submitted | 2018 | 147 | PD | 62.5 | 49.7 | No |  | No |
| OffRoad (Helmich) | In progress^c^ | 2018 | 30 | PD | 64.1 | 23.3 | No |  | No |
| ParkGame II | In progress^c^ | 2018 | 25 | PD | 64.9 | 32 | No |  | Yes |
| Vital@Home | In progress^c^ | 2019 | 25 | PD | 69 | 32 | No |  | No |
| PEARL-PD | Accepted | 2019 | 101 | PD | 65.5 | 50.5 | Yes | 99 | No |
| PERSPECTIVE | N^b^ | 2019 | 215 | PD | 67.9 | 27.0 | No |  | No |
| RISE-PD | O | 2019 | 14 | PD + AP | 67.7 | 57.1 | No |  | Yes |
| Parkinson-In-Toom (PIT) | In progress^c^ | 2019 | 54 | PD | 62.3 | 40.7 | Yes |  | No |
| JOBGRIP part A | P^b^ | 2019 | 24 | PD | 56.6 | 29.2 | No |  | No |
| VENI (Helmich) | Q^b^ | 2019 | 64 | PD | 62.6 | 29.7 | Yes | 100 | Yes |
| Effectiveness of boxing using kicking techniques | In progress^c^ | 2019 | 28 | PD | 63.7 | 50 | No |  | No |
| Consciousness coaching | In progress^c^ | 2019 | 39 | PD | 63 | 41,0 | No |  | Yes |
| Parkinson Support | R^b^ | 2020 | 10 | PD | 75.8 | 60 | No |  | Yes |
| OffRoad (Nonnekes) | In progress^c^ | 2020 | 75 | PD | 69.0 | 24.0 | No |  | No |
| PRIME | S^b^ | 2020 | 984 | PD + AP | 69.7 | 38.8 | Yes | 99 | Yes |
| JOBGRIP part B | P^b^ | 2020 | 20 | PD | 54.7 | 20 | No |  | No |
| STEPWISE Parkinson pilot | In progress^c^ | 2020 | 30 | PD | 63.9 | 43.3 | No |  | Yes |
| YOPD | In progress^c^ | 2020 | 50 | PD | 48.1 | 50 | No |  | No |
| Vibrating Socks | T^b^ | 2020 | 31 | PD | 66.2 | 12.9 | No |  | No |
| TOP | U | 2021 | 52 | PD | 67.5 | 19.2 | No |  | No |

N = number of participants; NMS = non-motor symptoms; PD = Parkinson’s disease; AP = atypical parkinsonism

^a^Weighted for study size

^b^Protocol

^c^Study is still ongoing, or the study has been completed and a manuscript is currently being written

A Aerts MB, Esselink RA, Abdo WF, Meijer FJ, Drost G, Norgren N, et al. Ancillary investigations to diagnose parkinsonism: a prospective clinical study. J Neurol. 2015;262(2):346-56.

B Munneke M, Nijkrake MJ, Keus SH, Kwakkel G, Berendse HW, Roos RA, et al. Efficacy of community-based physiotherapy networks for patients with Parkinson's disease: a cluster-randomised trial. Lancet Neurol. 2010;9:46-54.

C Van der Marck MA, Munneke M, Mulleners W, Hoogerwaard EM, Borm GF, Overeem S, et al. Integrated multidisciplinary care in Parkinson's disease: a non-randomised, controlled trial (IMPACT). Lancet Neurol. 2013;12:947-56.

D Sturkenboom IH, Graff MJ, Borm GF, Veenhuizen Y, Bloem BR, Munneke M, et al. The impact of occupational therapy in Parkinson's disease: a randomized controlled feasibility study. Clin Rehabil. 2013;27(2):99-112.

E van Rumund A, Aerts MB, Esselink RAJ, Meijer FJA, Verbeek MM, Bloem BR. Parkinson's Disease Diagnostic Observations (PADDO): study rationale and design of a prospective cohort study for early differentiation of parkinsonism. BMC Neurol. 2018;18(1):69.

F Sturkenboom IHWM, Graff MJL, Hendriks JCM, Veenhuizen Y, Munneke M, Bloem BR, et al. Efficacy of occupational therapy for patients with Parkinson's disease: a randomised controlled trial. Lancet Neurol. 2014;13(6):557-66.

G Strouwen C, Molenaar E, Munks L, Keus SHJ, Zijlmans JCM, Van den Berghe W, et al. Training dual tasks together or apart in Parkinson's disease: Results from the DUALITY trial. Mov Disord. 2017;32:1201-10.

H Nijhuis FAP, Elwyn G, Bloem BR, Post B, Faber MJ. Improving shared decision-making in advanced Parkinson's disease: protocol of a mixed methods feasibility study. Pilot Feasibility Stud. 2018;4:92.

I Hommel ALAJ, Meinders MJ, Weerkamp NJ, Richinger C, Schmotz C, Lorenzl S, et al. Optimizing Treatment in Undertreated Late-Stage Parkinsonism: A Pragmatic Randomized Trial. J Parkinsons Dis. 2020;10(3):1171-84.

J Silva de Lima AL, Hahn T, Evers LJW, de Vries NM, Cohen E, Afek M, et al. Feasibility of large-scale deployment of multiple wearable sensors in Parkinson's disease. PLoS One. 2017;12(12):e0189161.

K Damman OC, Verbiest MEA, Vonk SI, Berendse HW, Bloem BR, de Bruijne MC, et al. Using PROMs during routine medical consultations: The perspectives of people with Parkinson's disease and their health professionals. Health Expect. 2019;22(5):939-51.

L Bloem BR, Marks WJ, Jr., Silva de Lima AL, Kuijf ML, van Laar T, Jacobs BPF, et al. The Personalized Parkinson Project: examining disease progression through broad biomarkers in early Parkinson's disease. BMC neurology. 2019;19(1):160.

M Evers LJW, Raykov YP, Krijthe JH, Silva de Lima AL, Badawy R, Claes K, et al. Real-Life Gait Performance as a Digital Biomarker for Motor Fluctuations: The Parkinson@Home Validation Study. J Med Internet Res. 2020;22(10):e19068.

N PERSPECTIVE, Protocol. [Available from: <https://www.clinicaltrials.gov/ct2/show/NCT03963388?term=perspective&cond=parkinson&draw=2&rank=1>.

O van de Wetering-van Dongen VA, Nijkrake MJ, Koenders N, van der Wees PJ, Bloem BR, Kalf JG. Experienced Respiratory Symptoms and the Impact on Daily Life from the Perspective of People with Parkinson's Disease: A Grounded Theory. J Parkinsons Dis. 2022;12(5):1677-91.

P JOBGRIP, Protocol. [Available from: <https://www.trialregister.nl/trial/8015>.

Q VENI(Helmich), Protocol. [Available from: <https://doi.org/10.1186/ISRCTN89589002>.

R Lennaerts H, Groot M, Steppe M, van der Steen JT, van den Brand M, van Amelsvoort D, et al. Palliative care for patients with Parkinson's disease: study protocol for a mixed methods study. BMC Palliat Care. 2017;16(1):61.

S Ypinga JHL, Van Halteren AD, Henderson EJ, Bloem BR, Smink AJ, Tenison E, et al. Rationale and design to evaluate the PRIME Parkinson care model: a prospective observational evaluation of proactive, integrated and patient-centred Parkinson care in The Netherlands (PRIME-NL). BMC Neurol. 2021;21(1):286.

T VibratingSocks, Protocol. [Available from: <https://www.trialregister.nl/trial/7679>.

U van den Heuvel L, Meinders MJ, Post B, Bloem BR, Stiggelbout AM. Personalizing decision-making for persons with Parkinson’s disease: where do we stand and what to improve? Journal of Neurology. 2022;269:3569–78.

Supplementary material B. Association of time period with participant characteristics and study outcomes

|  | Proportion of females | | Mean age | | Proportion of native Dutch people | | Proportion which included atypical parkinsonism | | Proportion ethnicity reported | | Proportion NMS as outcome | |
| --- | --- | --- | --- | --- | --- | --- | --- | --- | --- | --- | --- | --- |
|  | W | p | W | p | W | p | W | p | W | p | W | p |
| Not weighted for study size |  |  |  |  |  |  |  |  |  |  |  |  |
| 2010-2015 vs. 2003-2009 | 14 | 0.76 | 14 | 0.76 | 0 | 1.00 | 13 | 0.88 | 13 | 0.88 | 11 | 0.89 |
| 2016-2021 vs. 2003-2009 | 51 | 0.76 | 55 | 0.56 | 2 | 1.00 | 54 | 0.38 | 46 | 1.00 | 42 | 0.74 |
| Weighted for study size |  |  |  |  |  |  |  |  |  |  |  |  |
| 2010-2015 vs. 2003-2009 | 13 | 0.56 | 14 | 0.41 | 0 | 1.00 | 11 | 1.00 | 11 | 1.00 | 9 | 0.88 |
| 2016-2021 vs. 2003-2009 | 12 | 1.00 | 15 | 0.61 | 2 | 1.00 | 12 | 1.00 | 9 | 0.49 | 9 | 0.57 |

NMS = Non-motor symptoms; W = Wilcoxon rank-sum test statistic, p = p-value. Proportions, proportion of studies.
